# Magnetic Resonance Imaging Guidance Mitigates the Effects of Intrafraction Prostate Motion During Stereotactic Body Radiotherapy for Prostate Cancer

**DOI:** 10.7759/cureus.2442

**Published:** 2018-04-06

**Authors:** John V Hegde, Minsong Cao, Victoria Y Yu, Amar U Kishan, Narek Shaverdian, James Lamb, Michael L. Steinberg

**Affiliations:** 1 Department of Radiation Oncology, University of California, Los Angeles

**Keywords:** stereotactic body radiotherapy (sbrt), prostate cancer, prostate motion, mri guidance, cine imaging

## Abstract

The accurate delivery of stereotactic body radiotherapy (SBRT) for definitive prostate cancer treatment is aided by intrafraction image guidance. The common methods for intrafraction imaging require the invasive placement of fiducial markers or electromagnetic transponders. Recently, a magnetic resonance imaging (MRI)-guided tri-cobalt-60 head radiotherapy system has become available for treatment, which can utilize real-time cine MRI to non-invasively track prostate motion. We report on a clinical vignette using this technique to deliver SBRT for the definitive treatment of intermediate-risk prostate cancer. The incorporation of an MRI-guided radiotherapy system and the implementation of real-time adaptive dose delivery accounting for intrafraction anatomic motion may improve outcomes using this technique.

## Introduction

Stereotactic body radiotherapy (SBRT) is being increasingly utilized as a definitive treatment option for prostate cancer [1]. This technique exploits the low alpha/beta ratio of prostate cancer cells to potentially improve efficacy while also improving patient convenience by condensing treatment length. One of the challenges with this treatment involves minimizing the impact of inter- and intrafraction target motion when only five fractions are being delivered. Several image guidance methods have been developed for intensity-modulated radiation therapy (IMRT) treatments in prostate cancer to account for prostate motion and deformation. These include cone beam computed tomography (CBCT), stereoscopic x-ray verification using intraprostatic fiducial markers, and the Calypso system (Varian Medical Sytems, Palo Alto, California, United States) using electromagnetic transponder placement.

Intrafraction prostate motion and deformation are particularly important with SBRT delivery given that more dose is delivered per day in many fewer fractions than in a conventionally fractionated radiation therapy course. Therefore, a significant deviation in even a single fraction may alter 20% of the delivered dose in a particular location, resulting in the over-dosing of normal structures or the under-dosing of the prostate. We report on a clinical vignette illustrating the opportunity for non-invasive, accurate treatment delivery of SBRT for prostate cancer with the magnetic resonance imaging (MRI)-guided tri-cobalt-60 radiation therapy system.

## Case presentation

A 74-year-old man presented with a newly diagnosed, favorable-intermediate risk prostate cancer (clinical T-category T1c, prostate-specific antigen (PSA) 5.54 ng/ml, biopsy Gleason score 3+4=7). He was initially found to have an elevated screening PSA, for which he was referred for a diagnostic prostate MRI exam. This revealed a 42-gram prostate with two suspicious lesions at the left anterior peripheral mid-gland, measuring 1.9 cm, and at the right anterior peripheral apical mid-gland lesion, measuring 0.9 cm. No evidence of an extraprostatic extension was seen, including either seminal vesicle invasion or neurovascular bundle involvement. No suspicious lymphadenopathy or bone lesions were appreciated. Subsequently, a targeted and limited systematic prostate biopsy was performed. This procedure was performed under anesthesia, given the patient’s extreme apprehension of needles. Pathology revealed three out of eight cores having evidence of malignancy, with two cores containing Gleason score 3+4=7 disease and one core containing 3+3=6 disease. 

The patient refused further invasive manipulation of his perineum as well as consideration of brachytherapy or radical prostatectomy as treatment options. His reticence for the invasive manipulation of his prostate also resulted in an active surveillance program incorporating further biopsies being an undesirable option. He was very interested in an external beam radiation technique to treat his prostate cancer, and he was most interested in SBRT given the convenience of the treatment.

**Figure 1:**
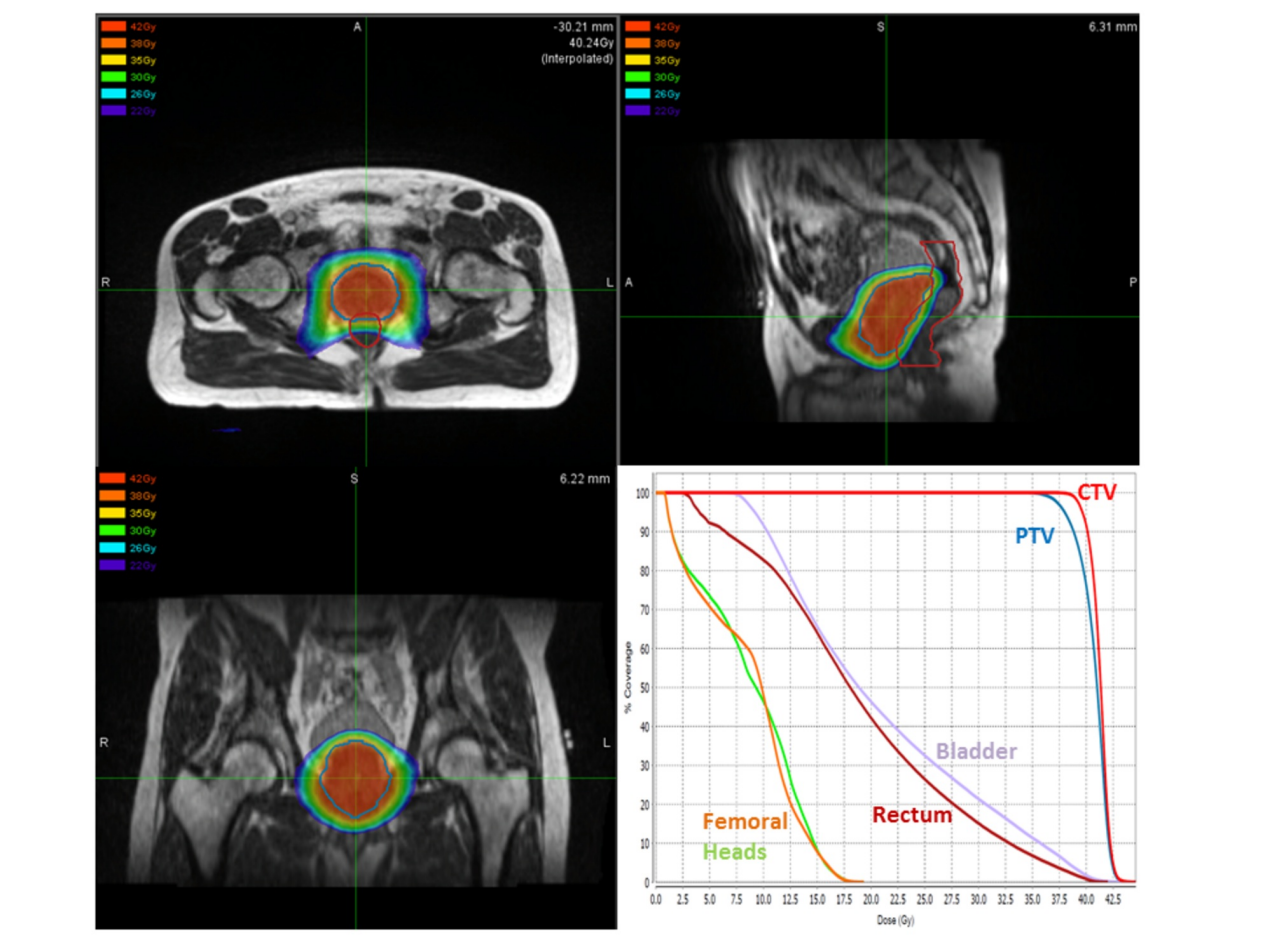
Prostate Stereotactic Body Radiotherapy Treatment Plan Dose Distribution and Dose-Volume Histogram Top-left, top-right, and bottom-left figures display the dose distribution of the prostate stereotactic body radiotherapy treatment plan in the axial, sagittal, and coronal planes, respectively. The bottom-right figure represents the dose-volume histogram of the treatment plan.

In order to accommodate his desires, it was decided to deliver SBRT utilizing the 0.35-Tesla MRI-guided tri-cobalt-60 radiotherapy system, which would obviate the need for fiducial placement. MRI- and computed tomography (CT)-based simulations were performed for radiation planning. A true fast imaging with steady-state free precession (TRUFI) sequence was performed to acquire the three dimensional MRI images with 1.5-mm isotropic spatial resolution and a field of view of 50x45x43 cm without contrast injection and a total acquisition time of 172 seconds. The MRI-based simulation was used for volumetric target and organs-at-risk (OARs) anatomic delineation. The CT-based simulation was utilized to provide electron density information for treatment planning. The prescribed dose to the planning target volume (PTV) was 38Gy in five fractions. The radiation dose distribution and dose-volume histogram (DVH) of the treatment plan are presented in Figure 1.

**Figure 2:**
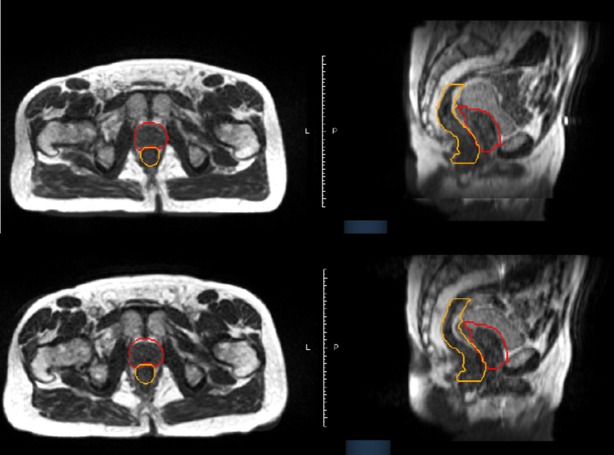
Multiplanar Imaging at Magnetic Resonance Imaging Simulation Is Consistent with Image Verification on the Day of Treatment Axial (left) and sagittal (right) imaging of the pelvis at magnetic resonance imaging (MRI) simulation (top) and on the day of treatment (bottom) are shown. The prostate planning target volume (PTV) is contoured in red, while the rectum is contoured in orange.

Treatment was administered every other day over the course of 10 days. MRI guidance was used for patient setup prior to treatment delivery (Figure 2).

Real-time cine MRI imaging at a rate of four frames/second was also utilized during treatment delivery to evaluate and gate radiation delivery based on intrafraction anatomic changes (Video 1).

**Video 1:** Intrafraction Imaging Reveals Significant Rectal Distension due to Bowel Gas Movement, Which Moves the Prostate Outside of the Planning Target Volume This time-lapse video (played at four times normal speed) of real-time magnetic resonance imaging (MRI) imaging of the pelvis in the sagittal plane during treatment shows a significant motion of the prostate (thin red line) relative to the treatment gating window (thick red line) as a result of bowel gas distending the rectum.

**Figure 3:**
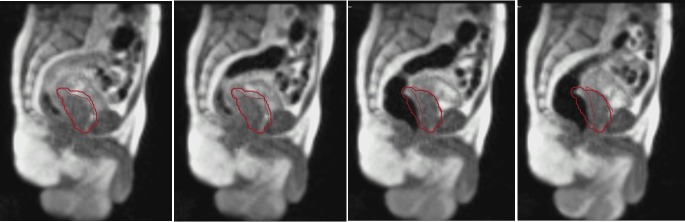
Sequential Image Capture Illustrating Prostate Motion due to Rectal Distension These sagittal images capture intrafraction prostate motion (contoured as the thin red line) away from the treatment gating window (thick red line) at various time points due to rectal distension from bowel gas movement.

A 3-mm gating margin from the prostate contour was set as the gating boundary. Beam delivery was halted if the prostate moved outside the gating boundary or if the system detected low image correlation between the acquired cine frames and the initially acquired three-dimensional images. Despite that, minimal interfraction motion was observed on daily MRI setup images. As shown in Figure 2, significant rectal volume deviations were found during treatment delivery due to bowel gas distending the rectum at the level of the prostate (Video 1 and Figure 3).

**Figure 4:**
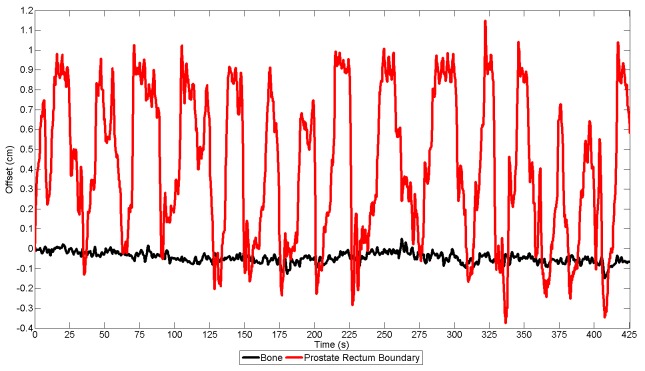
Tracking of the Prostate-Anterior Rectum Interface Reveals Large Intrafraction Deviations Caused by Intermittent Rectal Distension due to Bowel Gas Movement Two tracking points were placed on the prostate-rectal wall boundary (red) and pubic bone (black) on the cine magnetic resonance imaging (MRI) to track motion during treatment. Significant anterior-posterior deviation (up to 1.0 cm) was observed at the tracking point placed at the prostate-rectum boundary, indicating repetitive prostate movement caused by gas motion. As a control, the point placed on the pubic bone demonstrated minimal motion.

This resulted in significant intrafraction prostatic motion during every treatment fraction, even with bowel preparation recommendations including simethicone. Treatment was stopped either by the radiotherapy system or manually stopped by the treatment team to limit the treatment delivered when bowel gas was distending the rectum further into the PTV and the prostate out of the PTV. Treatment was resumed when the prostate returned to its desired position. When tracking the prostate-anterior rectum interface, up to 1 cm of anterior-posterior deviation was seen during treatment (Figure 4).

## Discussion

This clinical vignette presents one of the major challenges with SBRT delivery in prostate cancer – rectal distension leading to inaccurate treatment delivery. At our institution, linear accelerator-based prostate SBRT employs both CBCT and stereoscopic x-ray imaging for image guidance [1]. After the attending radiation oncologist evaluates the anatomy on CBCT prior to treatment delivery initiation, a verification of alignment with intraprostatic fiducial markers placed at the time of CT simulation is performed with stereoscopic x-ray imaging. The treatment is subsequently delivered using four half arcs with volumetric-modulated arc therapy (VMAT). Prior to every half arc being administered, stereoscopic imaging verification is performed to ensure optimal prostate alignment. Using this technique, our institution and others have reported little long-term difference in the quality of life analyses evaluating urinary and bowel domains as compared to prior to treatment [2].

However, there has been the suggestion of increased late toxicity when not utilizing similar image guidance techniques, in particular in the high-risk prostate cancer setting. This includes a Canadian prospective phase I/II study of prostate SBRT, which reported that 30% of men had grade two or greater gastrointestinal (GI) or genitourinary (GU) toxicity at six months [3]. Notably, patients in this study did not have fiducials placed or stereoscopic imaging during treatment delivery (only a CBCT prior to treatment). While nodal volumes were treated in this study, our institutional prospective phase II protocol of SBRT for high-risk prostate cancer, including nodal treatment, has found far more favorable toxicity, including late grade two GU and GI toxicity of 4.5% and 13.6%, respectively [4].

While prostate fiducial placement for the utilization of stereoscopic x-ray imaging appears to be a valuable technique for limiting the late toxicities of prostate cancer, there are situations in which fiducial placement may be contraindicated. For example, some patients may be unable to temporarily suspend anticoagulation for fiducial placement without an unacceptably high risk of thromboembolic events. Also, some patients may have an increased risk of infection due to comorbidities, including immunosuppression. Other patients may have already developed a serious infection following prostate biopsy, making further perineal manipulation with fiducial placement a less attractive option. In addition, some patients may desire a fully non-invasive treatment for prostate cancer.

In this case, our patient had a prohibitive fear of prostate fiducial placement. MRI-guided treatment with the tri-cobalt-60 head radiotherapy system provided a platform to administer radiation accurately in a completely non-invasive format. Also, this treatment provided further insight into the significant prostate motion that can occur during treatment. While this was known previously from studies performed with the Calypso system [5], this case report directly illustrates that intermittent rectal distension due to bowel gas may result in significant inaccuracies in dose delivery. While translational or rotational changes are appreciated with the Calypso system, prostate deformation is only directly evaluable with real-time MRI guidance. While our protocol is to perform stereoscopic localization after every half arc with our linear accelerator-based SBRT treatment, a significant deviation may be occurring during each half arc delivery. These deviations may be clinically significant in some patients, potentially resulting in the grade two toxicities seen with our own institutional protocol and the more significant late toxicities observed in the Canadian trial when no intrafraction imaging verification was used. Rectal distension may perhaps even result in an elevated local failure rate given the under-dosing of the PTV. Importantly, a rectal balloon has been reported to reduce intrafraction prostate motion [6-7], with hydrogel spacer performing similarly in a comparative study [8]. Either technique may serve to improve SBRT delivery, albeit with greater invasiveness added to the treatment.

Several limitations of this treatment protocol are important to note. First, some patients may not be candidates for MRI-based imaging with the tri-cobalt-60 head radiotherapy system, including those who are pacemaker-dependent or who have artificial hip instrumentation causing significant image distortions. Second, while cine MRI imaging may improve accurate treatment delivery, there are greater dosimetric limitations of tri-cobalt-60 head-based treatment delivery as compared to linear accelerator-based dosimetry, potentially limiting the improvement that this system may have for treatment delivery accuracy. An MRI-guided linear accelerator-based treatment delivery system may improve this limitation. Third, the use of MRI-based intrafraction imaging is time-consuming, as it extends the treatment delivery time when the treatment is halted due to rectal distension. As such, a more reliable real-time treatment adaptation of the delivery system to account for prostate motion is essential to optimize both treatment accuracy and efficiency. In addition, the spatial resolution of imaging is 1.5 mm, which results in a geometric distortion of less than 1 mm at a 10-cm radius from the isocenter and 1-2 mm at a 17.5-cm radius [9]. While encouraging, improvements in both spatial resolution and geometric distortion will aid in more accurate treatment delivery, which is especially important in SBRT treatment courses.

## Conclusions

SBRT is an increasingly common treatment for prostate cancer, and optimal image guidance is essential for accurate treatment delivery to maximize efficacy and minimize the toxicity profile. As an alternative to invasive fiducial or electromagnetic transponder placement, non-invasive real-time intrafraction cine MRI can optimize the accuracy of treatment delivery. The implementation of both MRI-guided radiation therapy and real-time dose delivery adaptation to prostate motion may improve the efficacy and efficiency of treatment delivery.
